# The Effect of Pore Directionality of Collagen Scaffolds on Cell Differentiation and In Vivo Osteogenesis

**DOI:** 10.3390/polym13183187

**Published:** 2021-09-20

**Authors:** Miguelangel Moncayo-Donoso, Gustavo A. Rico-Llanos, Diego A. Garzón-Alvarado, José Becerra, Rick Visser, Marta R. Fontanilla

**Affiliations:** 1 Tissue Engineering Group, Department of Pharmacy, Universidad Nacional de Colombia, Bogotá 571, Colombia; mmoncayod@unal.edu.co; 2 Biomimetics Laboratory, Biotechnology Institute, Universidad Nacional de Colombia, Bogotá 571, Colombia; dagarzona@unal.edu.co; 3 BIONAND, Andalusian Center for Nanomedicine and Biotechnology, University of Malaga, 29001-29018 Malaga, Spain; garico@bionand.es (G.A.R.-L.); becerra@uma.es (J.B.); 4 Networking Research Center on Bioengineering, Biomaterials and Nanomedicine, CIBER-BBN, 29001-29018 Malaga, Spain; 5 Department of Cell Biology, Genetics and Physiology, University of Malaga, IBIMA, 29001-29018 Malaga, Spain

**Keywords:** bone tissue engineering, osteogenesis, type I collagen, pore directionality, BMP-2, VEGF

## Abstract

Although many bone substitutes have been designed and produced, the development of bone tissue engineering products that mimic the microstructural characteristics of native bone remains challenging. It has been shown that pore orientation within collagen scaffolds influences bone matrix formation by the endochondral route. In addition, that the unidirectional orientation of the scaffolds can limit the growth of blood vessels. However, a comparison between the amount of bone that can be formed in scaffolds with different pore orientations in addition to analyzing the effect of loading osteogenic and proangiogenic factors is still required. In this work we fabricated uni- and multidirectional collagen sponges and evaluated their microstructural, physicochemical, mechanical and biological characteristics. Although the porosity and average pore size of the uni- and multidirectional scaffolds was similar (94.5% vs. 97.1% and 260 µm vs. 269 µm, respectively) the unidirectional sponges had a higher tensile strength, Young’s modulus and capacity to uptake liquids than the multidirectional ones (0.271 MPa vs. 0.478 MPa, 9.623 MPa vs. 3.426 MPa and 8000% mass gain vs. 4000%, respectively). Culturing of rat bone marrow mesenchymal stem cells demonstrated that these scaffolds support cell growth and osteoblastic differentiation in the presence of BMP-2 in vitro, although the pore orientation somehow affected cell attachment and differentiation. The evaluation of the ability of the scaffolds to support bone growth when loaded with BMP-2 or BMP-2 + VEGF in an ectopic rat model showed that they both supported bone formation. Histological analysis and quantification of mineralized matrix revealed that the pore orientation of the collagen scaffolds influenced the osteogenic process.

## 1. Introduction

Worldwide, millions of bone grafts are used for orthopaedical procedures each year to treat bone defects due to tumor resection, nonunion fractures, or osteonecrosis and to perform spinal fusions and maxillofacial or bone joint reconstruction [1,2]. Although the use of autografts is still considered the gold standard therapy and is used whenever possible, its limited availability and some associated disadvantages, such as donor site morbidity [3,4], have promoted research and development of bone substitutes based on bone tissue engineering (BTE). Tissue engineering is a field that has become increasingly important in biomedicine during the last decades and has been dedicated to the design of better and more sophisticated biomaterials for a great variety of applications [5,6]. In this context, the production of 3D porous scaffolds with significant similarity to the natural bone matrix has been a challenge for BTE researchers [7,8]. 

The bone matrix is mainly made of collagen type I and hydroxyapatite (HAp) crystals. When combined and arranged in a particular way, these components confer unique mechanical properties to the bone, making it stiff and flexible at the same time [9]. The microstructural organization of the extracellular matrix is different in cortical and trabecular osseous tissue. While the former is dense and solid, the latter shows a honeycomb-shaped network filled with bone marrow [10]. The structural unit of the compact cortical bone is the osteon, composed of cylindrical walls called lamellae, in which the collagen fibers run unidirectionally, oblique to the longitudinal axis of the osteon. Conversely, the lamellae within the trabecular bone are irregularly arranged and do not form osteons. Although the trabeculae have a poorly organized and random disposition, they align accordingly to lines of stress and help to distribute mechanical forces along the bone. The unique mechanical properties of bone tissue depend, in a significant part, on its organized microstructure [11]. Therefore, when BTE osteoconductive scaffolds are grafted into bone lesions, their microstructure will determine the mechanical properties of the new-formed bone before its natural remodeling.

Existing literature describing BTE scaffolds bioinspired by bone tissue architecture has shown that their microtopography can control cellular responses through scaffold-cell interactions that activate proliferation and osteogenic differentiation pathways [12]. The oriented microstructures of scaffolds guide the elongation and growth-orientation of the cells seeded on and into them. This feature can be particularly useful to promote the regeneration of unidirectional tissues such as peripheral nerves, tendons and muscles, among others [12]. Research that evaluates the performance of uni- and multidirectional scaffolds in bone repair/regeneration is still scarce. However, it is possible to claim that those mimicking the bone microstructural directionality are more osteoconductive and lead to greater mechanical strength than scaffolds that lack a predominant directionality. Moreover, scaffolds with unidirectional channels have shown higher infiltration of cells than those without this unidirectionality [13,14].

It has been demonstrated that the pore orientation of scaffolds can influence bone matrix formation. Cell- and growth factor free-collagen scaffolds with unidirectional porosity showed enhanced endochondral bone formation when implanted in femoral defects in rats [15]. This work also showed that the unidirectional orientation of collagen scaffolds could limit the growth of newly formed blood vessels. However, a comparison of the amount of bone formed within unidirectional and multidirectional scaffolds is still required, as well as analyzing the effect of adding growth factors related with the osteogenic process to this system. In the present work we have produced collagen type I scaffolds with unidirectional (UC) and multidirectional (MC) pores. In vitro, we analyzed their microstructural, physicochemical and mechanical properties, as well as their ability to support cell attachment, growth and differentiation. Since it has been well described that the osteogenic activity of bone morphogenetic proteins can be synergistically promoted by angiogenic factors such as VEGF [16,17,18], we evaluated the ability of these scaffolds to guide bone growth when loaded with BMP-2 or BMP-2 and VEGF in a rat ectopic model, as well as the effect that the implanted UC and MC scaffolds had on the amount of bone matrix formed.

## 2. Materials and Methods

### 2.1. Manufacturing of Uni- and Multidirectional Collagen Type I Scaffolds

Collagen type I (Col-I) was isolated from bovine fasciae as previously described [19], obtaining a 5 mg/mL colloidal dispersion. The collagen fibrils were cross-linked by the addition of 0.02% (*w*/*v*) glutaraldehyde and maintained in orbital agitation at 140 rpm for 24 h at room temperature. The Col-I scaffolds with multidirectional porosity (MC) were obtained by freezing the cross-linked Col-I dispersion at −20 °C for 24 h. Scaffolds with unidirectional porosity (UC) were obtained by modifying and up-scaling a previously standardized procedure [20]. Briefly, the Col-I dispersions were cast in molds that had one side covered by a metallic material and, in turn, had one end dipped in liquid nitrogen to create a temperature gradient with an axial orientation. The system described above was placed in an insulated container that kept the temperature of the enclosed air between 0 and 4 °C, which is important to avoid a multidirectional freezing. In this way, the ice crystals grew from the coldest (−196 °C) side of the mold towards the opposite side. This device also controlled the freezing speed and time (6 h). The frozen MC and UC scaffolds were lyophilized (Virtis, SP Industries, Warminster, PA, USA) for 48 h and sterilized with ethylene oxide. For all the further experiments, circular pieces (5 mm diameter and 2 mm thick) randomly obtained from 5 × 5 cm sheets were used.

To analyze if any residual glutaraldehyde was left on the final sponges, samples (1 cm^2^) of MC and UC scaffolds were incubated (60 °C, 12 h) in 1 mL of a solution containing 1 M NaOH, 0.1 M glycine and 0.1 M Na_2_SO_3_ and centrifuged (400× *g*, 5 min). Afterwards, 100 µL of the supernatant was transferred to a 96-well plate. The absorbance of the samples was measured at 238 nm (TRIAD Multimode Detector, Dynex, Chantilly, VA, USA). The readings were interpolated on a calibration curve prepared with known concentrations of glutaraldehyde. Experiments were done in triplicate.

### 2.2. Scaffold Topography and Microstructure

Several microstructural characteristics of the scaffolds, such as pore shape, pore interconnectivity and microtopography, were evaluated by Environmental Scanning Electron Microscopy (ESEM; FEI Quanta 200; FEI, Hillsboro, OR, USA), taking images from three randomly selected fields at three different sites of each MC and UC scaffold. The acquisition parameters used were: 10.0 KV, spot = 3.0 and pressure = 70 Pa. 

### 2.3. Porosity and Pore Size

The percentage of porosity (P) of the scaffolds was calculated as the ratio between the apparent density of the scaffold and the theoretical density of collagen. Randomly selected samples of each scaffold (*n*-3) were dried at 60 °C for 24 h. The bulk density was calculated from the mass of the dry scaffold and its hydrated volume, which was obtained using a digital balance (±0.0001 g) and a digital calibrator (±0.01 mm), respectively. The theoretical density of collagen was assumed to be 1.23 g/cm^3^, according to [21]. 

Pore size was evaluated using hydrated, 100 μm-thick pieces of each scaffold type (*n*-3), which were observed under a phase-contrast inverted microscope (Eclipse TS100 - Sight DS-2Mv, Nikon, Tokyo, Japan). A total of 100 pores per scaffold were analyzed using the ImageJ v.1.51u software (Wayne Rasband, NIH, Bethesda, MD, USA). In this work, cylindrical pores were not seen in the UC and MC scaffolds. In cross-sectional images of the pores analyzed, the major and minor axes (*a* and *b*, respectively) of the ellipse that best fit each pore wall were measured. The formula used included a correction factor (1.5) to correct the effect of those pores that were not cut perpendicular to the axis of the ellipse [22,23]. Pore size (PS) was calculated as:

(1) $$\mathrm{PS}=1.5\times 2\times [\frac{\sqrt{a^{2}+\;b^{2}}}{2}]$$

### 2.4. Mechanical Testing 

Tensile testing was performed using a Universal Machine for Mechanical Test AGS-X (Stable Micro System, Surrey, UK) equipped with a 50 kN load cell. Rectangular dried “dog bone-shaped” pieces of UC and MC scaffolds (*n*-3) were stretched at a rate of 1 mm/min at room temperature and the values for the elasticity modulus, maximum elongation and breaking point at tension were collected. The tensile strength was also measured in samples swollen as described in 2.7. The stretching of the UC scaffolds was done in the direction of the oriented pore channels. Experiments were carried out in triplicate.

### 2.5. Biodegradability Test

The biodegradability of the scaffolds was evaluated by measuring their degradation rate when exposed to the enzymatic activity of type I collagenase. Briefly, samples with a dry weight of 1.0 mg (*n*-3 for each time point) were first dried at 37 °C for 3 h and then incubated at 37 °C in 1 mL of 50 mM CaCl_2_, 0.1 M Tris-HCl, pH 7.4 for 30 min. Afterward, 2 mL of Tris-HCl-collagenase solution (Sigma-Aldrich, St. Louis, MO, USA) was added to reach a final collagenase concentration of 1 mg/mL (125 CDU). The scaffolds were kept at 37 °C with gentle stirring for 24, 48, 72 or 96 h. The scaffolds were immersed in 0.5 mL of 0.25 M EDTA and kept on ice to stop the enzymatic action at each time point. Then, they were washed three times with distilled water for 10 min each and dried at 37 °C for 3 h. Finally, they were weighed and the percentage of degradation (D) was calculated. Experiments were carried out in triplicate.

### 2.6. Fourier Transform Infra-Red Spectroscopy (FT-IR)

Three scaffolds of each type, with an approximate weight of 2.0 mg, were used. FT-IR spectra were obtained using a Jasco 4200 spectrophotometer (Jasco, Tokyo, Japan), equipped with an attenuated total reflectance cell (ATR), in a spectral interval from 400 to 4000 cm^−1^ and with a resolution of 4 cm^−1^, averaging 4000 scans.

### 2.7. Swelling

A described procedure [24] was modified to measure the swelling behavior of the scaffolds. For this purpose, circular samples of the scaffolds (5 mm diameter and 2 mm thick) were dried (37 °C, 48 h) and weighed. Afterward, they were immersed in 1 mL of PBS (pH 7.4) and incubated at 37 °C. Every 5 min for the first 20 min and later at 30, 60, 120 and 180 min, three scaffolds were removed from the PBS and placed on a metallic net for 1 min, allowing the excess of PBS to drain until no further dripping was observed. The wet scaffolds were then weighed to calculate the percentage of mass gain.

### 2.8. Cells and Recombinant Proteins

Bone marrow-derived mesenchymal stem cells (rBM-MSCs) were isolated from 8-weeks old male Wistar rats (Charles River Laboratories, Écully, France). Briefly, the animals were anesthetized with a medetomidine/ketamine cocktail and sacrificed by cervical dislocation. Their femora were excised, the epiphyses were removed and the bone marrow was flushed out of the diaphysis with α-MEM (Sigma-Aldrich), passing them several times through a 21 G needle. Erythrocytes were removed by incubating the sample for 10 min in red blood cell lysing buffer (Sigma-Aldrich). The remaining cells were expanded as monolayers in T-75 culture flasks (Nunclon, Nunc, Roskilde, Denmark) in α-MEM supplemented with 10% fetal bovine serum (FBS), 2.5 mM L-glutamine, 100 U/mL penicillin, 100 μg/mL streptomycin and 1.25 µg/mL amphotericin, with a change of medium at day three to remove the non-adherent cells. The cells used for the in vitro assays were at passage 3–4.

The rhBMP-2 and rhVEGF-165 produced in CHO and SF21 cells, respectively, were purchased from R&D Systems (Minneapolis, MN, USA) and handled according to the manufacturer’s instructions.

### 2.9. Cell Attachment and Proliferation on the Collagen Scaffolds

To assess the ability of cells to attach to the collagen discs, each scaffold (5 mm diameter, 1–2 mm height) was placed in a 2 mL tube with 2.5 × 10^4^ cells in 1 mL of α-MEM supplemented with 10% FBS and incubated (3 h, 37 °C) with constant rolling. Afterward, the seeded scaffolds were transferred to wells of a 48-well culture plate and incubated overnight in α-MEM supplemented with 10% FBS. The next day (day 0), the cells were stained with CellTrackerTM Green (Thermo Fisher, Waltham, MA, USA) for 45 min, following the manufacturer’s recommendations. Afterward, they were visualized with a fluorescence stereoscope (*n*-3). Similar discs of a commercial hemostatic collagen type I sponge (Helistat^®^, Integra LifeScience, Billerica, MA, USA) were used as a control as previously described [25]. 

To determine the biocompatibility of the scaffolds and the ability of the cells to grow on them, other sets of discs (*n*-3) were seeded as previously described and kept in α-MEM with 10% FBS (37 °C, 5% CO_2_). At different time points (0, 3, 7 and 10 days), the number of cells on a set of scaffolds was evaluated using an MTT assay (Cell growth determination kit, Sigma-Aldrich). After solubilizing the formazan crystals produced within metabolically active cells with isopropanol, the absorbance at 520 nm of the solubilization solution was read with a plate spectrophotometer. The number of cells was calculated by interpolating within a standard curve obtained with known amounts of the same cells. 

### 2.10. Cell Differentiation on the Collagen Scaffolds

Collagen discs (5 mm diameter, 1–2 mm height) seeded with 5 × 10^4^ cells were used to assess the ability of the cells to differentiate towards the osteogenic lineage. Samples were incubated (37 °C, 5% CO_2_) in α-MEM supplemented with 2% FBS and with either 200 ng/mL rhBMP-2, 20 ng/mL VEGF or both growth factors combined (*n*-6 for each condition). After 10 days, the alkaline phosphatase (ALP) activity of the cells was measured using a *p*NPP-based method (SigmaFastTM *p*-nitrophenyl phosphate tablets, Sigma-Aldrich). Briefly, the medium was removed from the cells and the sponges were carefully washed twice with 200 µL of PBS and, finally, 100 µL of *p*NPP solution was added to each scaffold. After 20 min of incubation, the reaction was stopped with 100 µL of 1M NaOH and the absorbance of the resulting solution was read at 405 nm in a plate spectrophotometer.

### 2.11. In Vivo Ectopic Osteogenesis Assay

General animal care and surgical procedures were carried out following the Spanish (RD53/2013) and International (Directive 2010/63/EU) legislation concerning the protection of animals for scientific research purposes and were approved by the bioethics committee of the Andalusian Center for Nanomedicine and Biotechnology (BIONAND), Spain, where the experiments were performed. A total of twenty-three 10-week-old male Wistar rats (Charles River Laboratories, Écully, France) were used for this study.

For histological analysis, the scaffolds were prepared by loading collagen discs (5 mm diameter, 1–2 mm height) with 10 µL of a solution containing 500 ng of rhBMP-2, 50 ng of rhVEGF-165, or both combined. As a negative control, a set of scaffolds was loaded with vehicles only. We knew from previous studies that 500 ng of rhBMP-2 is slightly above the minimum dose necessary to trigger ectopic osteogenesis in this system [26]. The sponges were allowed to dry for approximately 1 h in a laminar flow cabinet before their implantation.

The animals received preoperative analgesia and were anesthetized by sevoflurane inhalation. Their dorsal region was shaved and disinfected and incisions were made in the skin to implant the sponges into muscular pockets made in the dorsal muscles, which were closed with a single suture with non-absorbable braided silk. Each animal received four different implants (*n*-4 for each experimental group). After the surgery, the animals were housed individually with unlimited access to food and water for 21 days. At this time point, the animals were euthanized by CO_2_ inhalation and the implants were collected. They were placed in 4% buffered formaldehyde for 24 h and decalcified in Histofix^®^ Decalcifier 2 (PanReac Applichem, Barcelona, Spain) for 4 h. Afterward, the implants were dehydrated and included in paraffin for cutting 7 μm sections using a microtome. Hematoxylin-eosin, alcian blue and Masson-Goldner trichrome staining of the sections were used for their visualization with a conventional light microscope.

To quantify the expression of rSp7 transcription factor (osterix) another group of animals was used to implant collagen scaffolds as described above (*n*-4 for each experimental group). In this case, however, no scaffolds with only rhVEGF-165 were included in the study as it was already demonstrated that VEGF alone is not capable of inducing ectopic osteogenesis in this system. Twenty-one days after their implantation, the scaffolds were recovered and immediately frozen in liquid nitrogen. They were afterward minced using a mortar and pestle and total RNA was isolated using a commercial kit (NZY Total RNA Isolation kit, Lisbon, Portugal). 1 µg of each RNA sample was retrotranscribed using the PrimeScript™ RT Master Mix (Takara, Kusatsu, Japan) following the manufacturer’s instructions. The qPCR was performed using TB Green^®^ Premix Ex Taq™ (Takara) in a CFX-96 Real-Time PCR detection system (Bio-Rad, Hercules, CA, USA). Reactions were prepared with 10 ng of cDNA and 250 nM of forward and reverse primers, using the following thermal protocol: activation at 95 °C for 30 s and 40 amplification cycles (denaturation for 5 s at 95 °C and annealing/extension for 30 s at 60 °C). A melt curve, with 0.5 °C increments from 65 to 95 °C, was performed following amplification to analyze the generated products. The primers for SP7 and for the reference gene glyceraldehyde 3-phosphate dehydrogenase (Gapdh) were designed using the online Primer Blast Software (NIH; SP7 fwd: GATATGTCCCATCCCTACGG; SP7 rev: GCCCACCACCTAACCAAT; rGapdh fwd: CATGCCGCCTGGAGAAAC; rGapdh rev: CCCAGGATGCCCTTTAGT). Two technical replicates of every biological sample were included in the analysis. Expression of the target gene was normalized according to the Gapdh reference gene using the ΔΔCt method.

To determine the amount of mineralization within the implants through the quantification of calcium, a third group of animals was used to implant collagen scaffolds as described earlier (*n*-10 for each experimental group). As for the qPCR analysis, no scaffolds with only rhVEGF-165 were included in the study. Twenty-one days after their implantation, the scaffolds were recovered and washed with 0.9% NaCl to remove the excess of erythrocytes. Each implant was then incubated for 24 h at room temperature in 10% HCl (*v*/*v*) for solubilizing the calcium minerals they contained. Finally, 10 µL of each sample were taken to measure their calcium content using an *o*-cresolphtalein complexone-based colorimetric method (Calcium OCC Kit, Linear Chemicals, Barcelona, Spain). This is an easy method for quantifying the amount of mineralized matrix within an implant without having to perform histomorphometric analyses, which can easily underestimate the amount of mineralized tissue.

### 2.12. Statistical Analysis

A normality analysis was performed using the Shapiro–Wilk test. Data were reported as the arithmetic mean ± standard deviation (SD). A one-way analysis of variance (ANOVA) and the Tukey post-hoc test were used to determine the significant differences among groups. The differences were considered significant with *p* < 0.05. Statistical analysis was performed using SPSS^®^ 25 software (IBM, Endicott, NY, USA).

## 3. Results

### 3.1. Scaffold Topography and Microstructure

**Figure 1:**
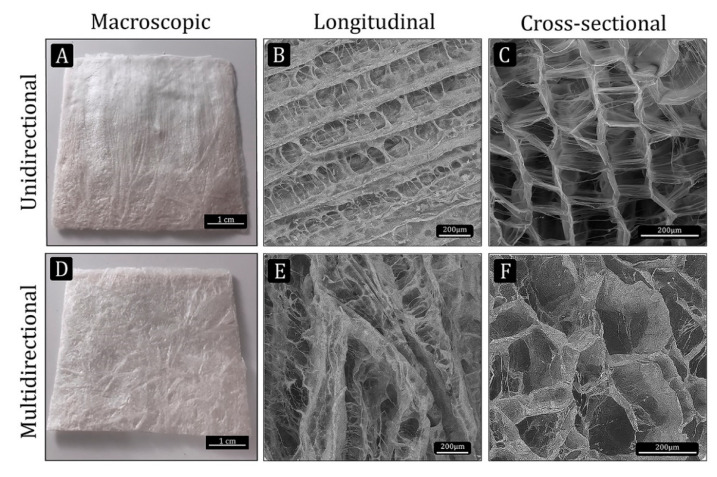
Macro- and microstructure of the collagen scaffolds manufactured by the freeze-dry methods. (**A** and **D**) macroscopic view of the unidirectional (UC) and multidirectional (MC) scaffolds, respectively (scale bar: 1 cm). (**B**,**C**,**E** and **F**) ESEM images showing the microstructural characteristics of UC and MC scaffolds (scale bar: 200 µm).

Samples for the different in vitro and in vivo analyses were cut from UC and MC collagen sheets (5 × 5 × 0.2 cm^3^). The macroscopic observation of the scaffolds already reflects the differences in the disposition of the collagen fibers between both materials (Figure 1A,D).

The microstructural characteristics of the UC and MC scaffolds were observed by ESEM. While a very organized microstructure could be seen in the UC scaffolds, the MC scaffolds displayed a more disorganized microstructure. The longitudinal view of UC showed the unidirectional channels separated from each other by thin walls, which in turn are connected by pores (Figure 1B). In MC, the channels formed by ice crystals during the non-controlled freezing run in several directions (Figure 1E). The cross-sectional images show the morphology of the pores and their interconnectivity (Figure 1C,F).

### 3.2. Porosity, Pore Size and Pore Orientation

**Figure 2:**
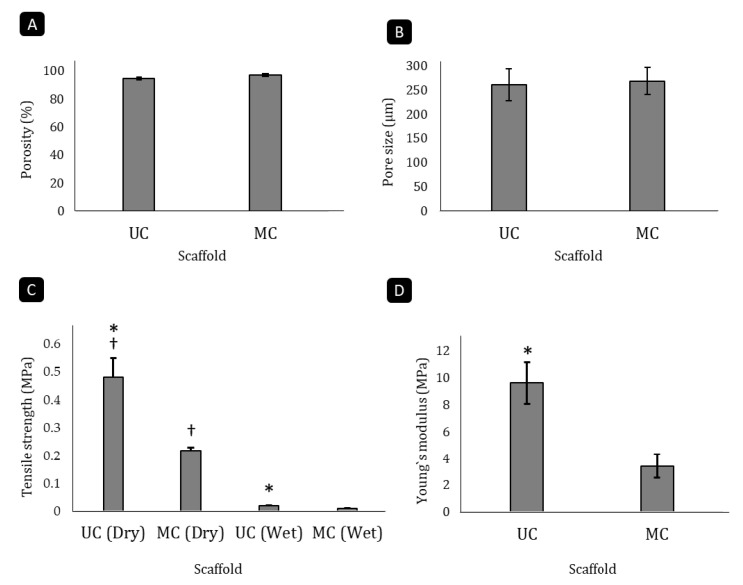
Analysis of microstructural and mechanical parameters of the scaffolds. Porosity (**A**), average pore size (**B**), tensile strength (**C**) and Young’s modulus (**D**) of the UC and MC scaffolds. Mean ± SD. *n*-3. * denotes differences between UC and MC scaffolds (*p* < 0.05), † denotes differences before and after swelling (*p* < 0.05).

The analysis of collagen scaffolds revealed that they were both highly porous. Although the unidirectional disposition of the channels in UC resulted in a slight reduction of the porosity in these scaffolds (94.5%) in comparison to the MC scaffolds (97.1%), this difference was not significant (Figure 2A). The measurement of the pore diameter using the linear intercept method showed that UC and MC had a very similar average pore size (260 µm and 268 µm, respectively; Figure 2B). A previous work [18] already evaluated the pore orientation of unidirectional and multidirectional type I collagen scaffolds according to described methodology [22,27,28]. These authors calculated the orientation index (OI) of collagen fibers within the multidirectional scaffolds, obtaining an OI close to zero, typical for random orientations, while it was close to 1.0 for the unidirectional scaffolds, which indicated that the pores were oriented toward the longitudinal axis.

### 3.3. Biomechanical Analysis of the Scaffolds

The pore directionality of the scaffolds influenced their biomechanical features. The UC scaffolds had a significantly higher tensile strength than the MC scaffolds in both the unswollen and swollen states (*p* < 0.05). When dry, the MC sponges could withstand a tensile load of 0.271 MPa before breaking, while the UC scaffolds resisted up to 0.478 MPa, which is more than double the amount (Figure 2C). In both cases, liquid uptake reduced the tensile strength around 95%. Furthermore, pore directionality significantly (*p* < 0.05) affected the Young’s modulus as well (Figure 2D), as the modulus of elasticity of the UC (9.623 MPa) was 2.8 times higher than that of the MC (3.426 MPa).

### 3.4. Biodegradability

**Figure 3:**
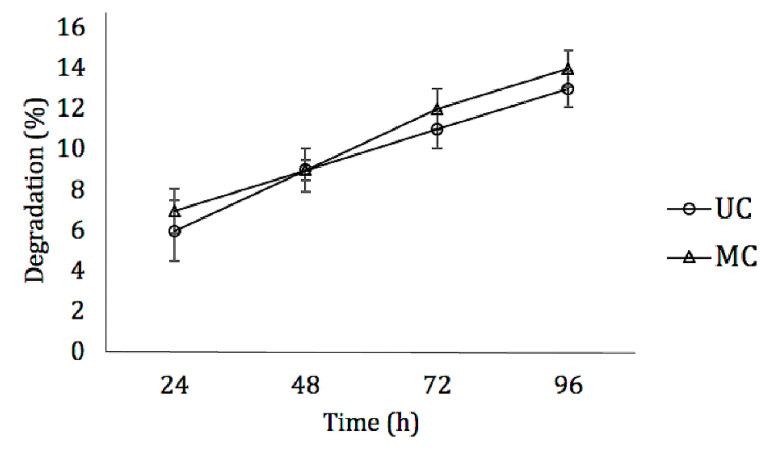
Degradation dynamics of UC and MC in the presence of type I collagenase. Mean ± SD; *n*-3.

No significant differences could be found between the degradation rate of UC and MC scaffolds when incubated with type I collagenase. During four days in the enzymatic solution, in both cases, the amount of degradation recorded was less than 15% (Figure 3).

### 3.5. Fourier Transform Infra-Red Spectroscopy (FT-IR)

**Figure 4:**
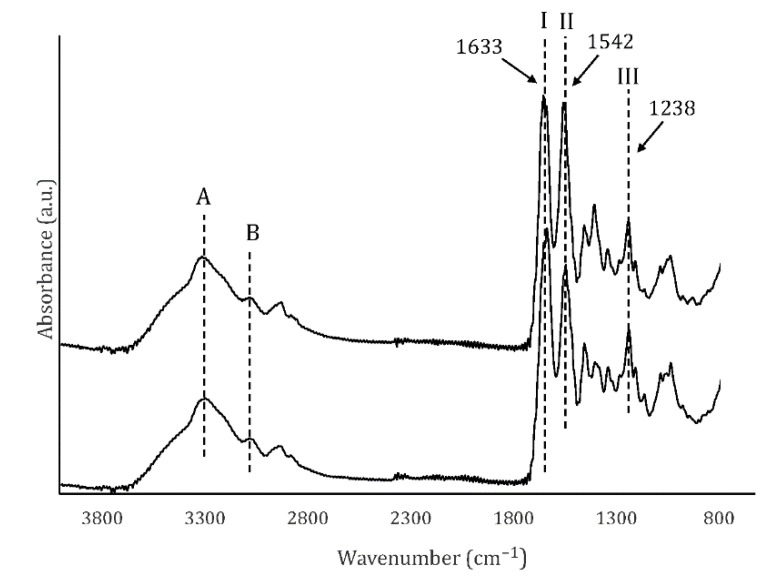
FTIR spectra of UC and MC. Amides A and B, as well as amides I, II and III (arrows) are shown.

The normalized FT-IR spectra of UC and MC scaffolds are shown in Figure 4. The samples showed the characteristic signals of collagen: amide A (N–H stretching) at ~3326 cm^−1^, amide B (N–H stretching) at ~3098 cm^−1^, amide I (C=O stretching) 1633 cm^−1^, amide II (C–N stretching, N–H bending) at 1542 cm^−1^ and amide III (C–N stretching, N–H deformation) at 1238 cm^−1^. The absorbance ratio of the amide III and 1450 cm^−1^ bands (AIII/1450 cm^−1^) was 0.853, what confirms that the collagen I triple helix structure was preserved during the processing of the scaffolds. According to previous works, this ratio is close to 1.0 in native collagen, while in denatured collagen (gelatin) it is close to 0.6 [29].

### 3.6. Swelling

**Figure 5:**
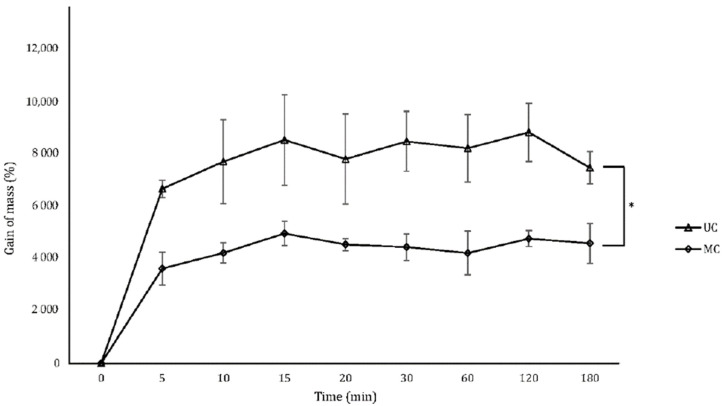
Gain of mass of the UC and MC scaffolds when swollen by PBS uptake. Mean ± SD; *n*-3; * *p* < 0.05.

Scaffolds were immersed in PBS until completely swollen to determine their capacity to uptake liquids. Both types of scaffolds reached their state of maximum liquid uptake after 10 min (Figure 5). The amount of PBS incorporated into the UC scaffolds led to an average mass gain of ≃8000% and was significantly higher than the amount that MC can contain, which average mass gain was of only ≃4000%.

### 3.7. Cell Attachment and Proliferation on the Collagen Scaffolds

**Figure 6:**
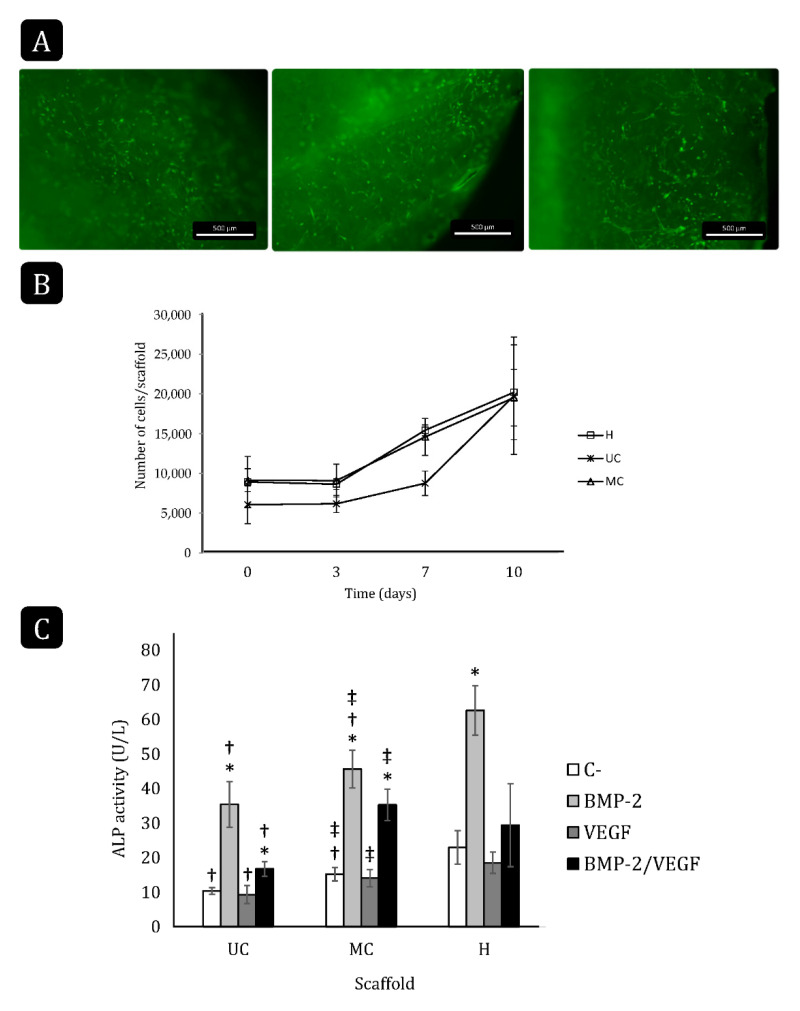
rBM-MSC attachment, growth and differentiation on the different collagen scaffolds. (**A**) Fluorescence stereoscopy images of the cells labeled with Cell Tracker Green and seeded on the scaffolds (scale bar: 500 µm). (**B**) Cell proliferation on the different scaffolds for 10 days. H: Helistat^®^; UC: unidirectional collagen; MC: multidirectional collagen. Mean ± SD; *n*-3. (**C**) Total ALP activity of rBM-MSCs seeded and cultured on UC, MC and Helistat^®^ for 10 days. * comparison of the treatments of each group with their respective negative control (C-); † comparison of groups with Helistat^®^ (H); ‡ comparison between UC and MC. Mean ± SD; *n*-6; *,†,‡ *p* < 0.05.

The residual glutaraldehyde within both types of scaffolds was found to be below 80 ng/mg of scaffold. To further test the biocompatibility of the collagen scaffolds and the ability of rBM-MSCs to adhere and grow on/inside them, cells were allowed to attach to the samples, including a commercial hemostatic collagen scaffold (Helistat^®^, H) as a control. Staining of the cells with *Cell Tracker Green* revealed a positive interaction with all the biomaterials as the cell bodies became elongated and they emitted numerous cytoplasmatic prolongations (Figure 6A).

There were no significant differences in seeding efficiency among the different collagen scaffolds, although the number of attached cells on UC was lower on average (≃6000 cells/scaffold) than on MC and on Helistat^®^ (≃9000 cells/scaffold; Figure 6B). In all three cases, cell proliferation could be observed after day three. Although the cells on UC started proliferating at a slower rate than on MC and Helistat^®^ between days three and seven, this rate was accelerated afterward and all the scaffolds reached a final cell density of ≃20,000 cells/scaffold at day 10.

### 3.8. Cell Differentiation on the Collagen Scaffolds

The ability of the rBM-MSCs to differentiate toward the osteogenic lineage when growing on the collagen scaffolds was evaluated in the presence of 200 ng/mL rhBMP-2, 20 ng/mL rhVEGF-165, or both for 10 days. As expected, the treatment with BMP-2 significantly increased the ALP activity when the cells were cultured on either scaffold compared to their respective controls (Figure 6C). Contrarily, VEGF did not increase the ALP activity and even counteracted the effect of BMP-2 on all three scaffolds. The control cultures, as well as those treated with BMP-2 only, had the highest ALP activity when growing on the Helistat^®^ scaffolds, followed by those growing on MC, while the ALP activity was the lowest when cells were seeded on the UC scaffold.

### 3.9. In Vivo Ectopic Osteogenesis

**Figure 7:**
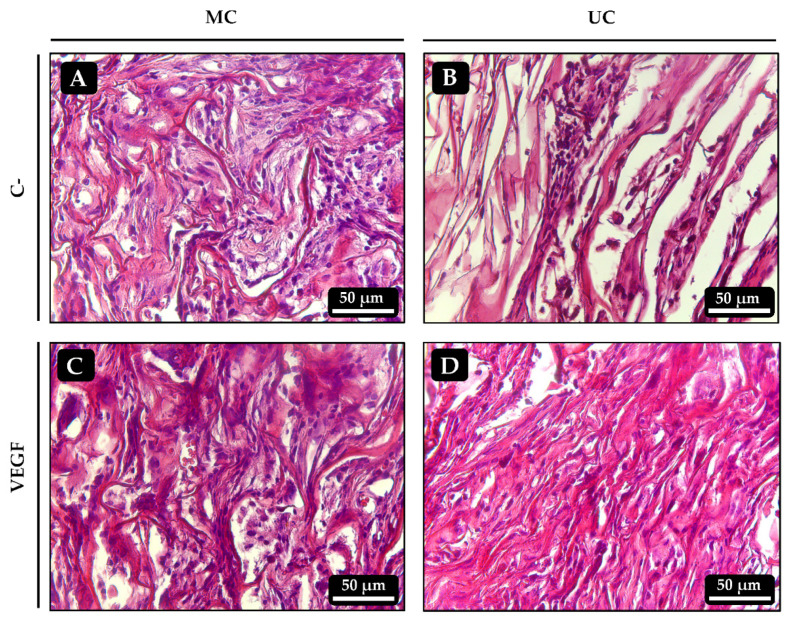
Histological sections of the ectopic implants stained with hematoxylin-eosin 21 days after implantation. (**A**,**B**) Control UC and MC scaffolds, respectively, loaded with vehicle only. (**C**,**D**) UC and MC scaffolds, respectively, loaded with 50 ng of VEGF.

Implants of UC and MC were prepared, loaded with BMP-2, VEGF or both and inserted into muscular pockets in rats for 21 days to evaluate the extent to which the different collagen scaffolds guide osteogenesis. After 21 days, none of the implants showed signs of inflammation (swollen tissues, hypervascularization or presence of giant multinucleated cells) or fibrous encapsulation. As expected, no chondro- nor osteogenesis could be observed in any of the control scaffolds, which showed varying degrees of cell infiltration and some small blood vessels (Figure 7A,B). Similarly, no bone tissue could be found within the implants that had been loaded with VEGF alone (Figure 7C,D).

**Figure 8:**
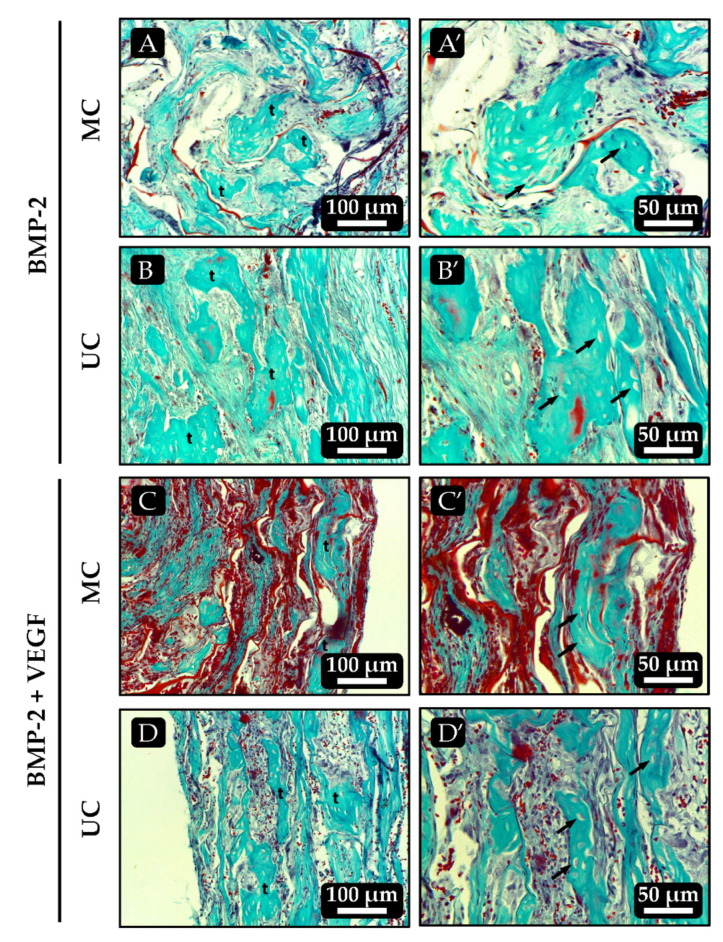
Histological sections of the ectopic implants stained with Masson-Goldner trichrome 21 days after implantation. (**A**,**B**) MC and UC scaffolds, respectively, loaded with 500 ng of BMP-2 only. (**C**,**D**) UC and MC scaffolds, respectively, loaded with 500 ng BMP-2 + 50 ng VEGF. Images on the right, denoted as X’, are higher magnifications of the images on the left. t: bone trabeculae; arrows: osteocytes.

**Figure 9:**
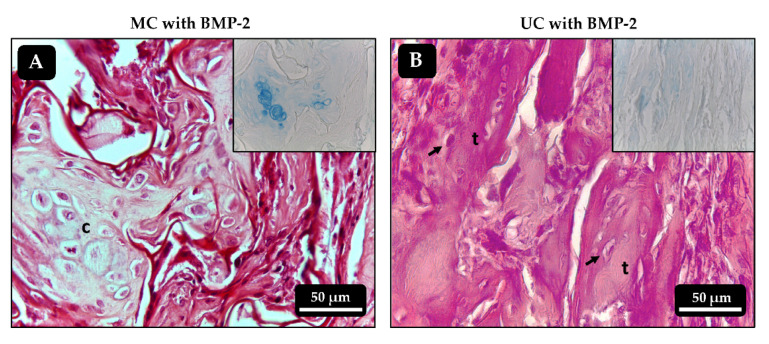
Histological sections of the ectopic implants stained with hematoxylin-eosin 21 days after implantation. (**A**,**B**) MC and UC scaffolds, respectively, loaded with 500 ng of BMP-2 only. Inserts correspond to alcian blue staining, highlighting cartilaginous formations in A. c: chondrocytes; t: bone trabeculae; arrows: osteocytes.

In most implants that had been loaded with BMP-2, either alone or combined with VEGF, bone tissue developed to varying extents. All the UC implants loaded with BMP-2 alone showed bone trabeculae that contained mature osteocytes and, in some of the samples most of these trabeculae run parallel to each other and to the unidirectional channels of the scaffold (Figure 8B and Figure 9B). Most of the MC implants loaded with BMP-2 alone also showed some signs of osteogenesis although, in this case, the bone trabeculae seemed to be slightly less mature, as areas of cartilage-to-bone transition could still be identified and they did not follow a clear spatial organization (Figure 8A and Figure 9A). Again, no clear effect of VEGF on angiogenesis could be seen in the scaffolds loaded with the combination of growth factors as they all very much resembled those loaded with BMP-2 alone. However, the spatial distribution of the bone trabeculae became more evident within the UC sponges loaded with BMP-2 + VEGF, as they showed a clear orientation parallel to the longitudinal axis of the implant (Figure 8D). As happened with the scaffolds loaded with BMP-2 only, the amount of bone tissue found within the MC scaffolds loaded with BMP-2 + VEGF was apparently lower and mainly limited to the most external areas of the implants (Figure 8C).

**Figure 10:**
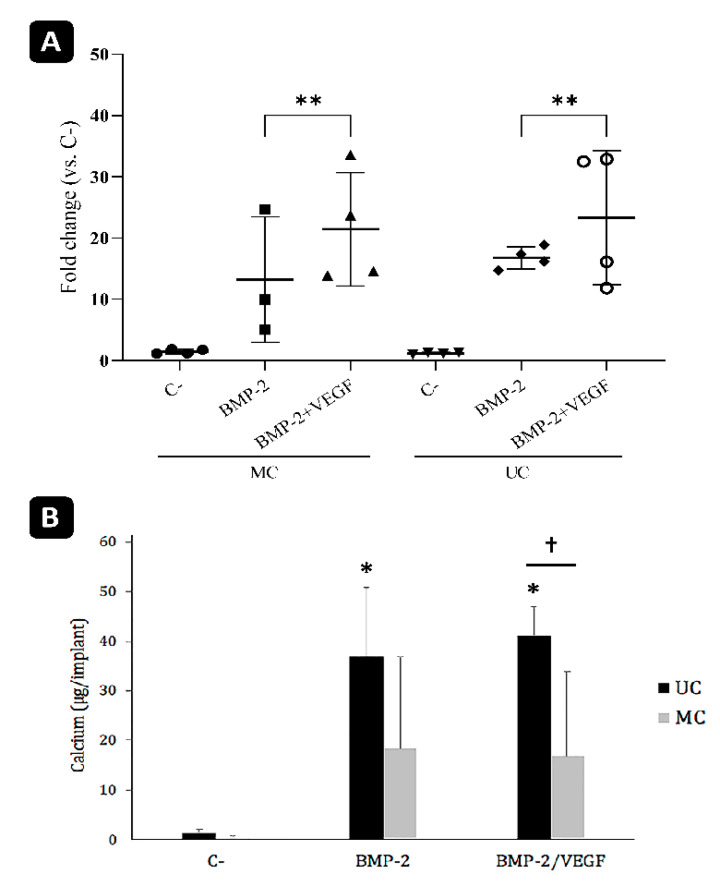
Analysis of the ectopic implants. (**A**) mRNA levels of rSp7 transcription factor (osterix) analyzed by qPCR. *n*-4. ** *p* < 0.01. (**B**) Calcium content of the ectopic bone formed within the graft 21 days after implantation. * comparison of the treatments of each group with their respective negative control (C-); †: comparison within each treatment. Mean ± SD; *n*-10; *, † *p* < 0.05.

The qPCR analysis revealed no significant differences between the transcription of rSp7 at day 21 post-surgery in the MC and UC implants (Figure 10A). Nevertheless, the positive effect of adding VEGF to the BMP-2 in both types of scaffolds was more clearly evidenced. On the contrary, the amounts of calcium measured within the implants as an indicator of matrix mineralization were consistent with the histological observations. As was expected, no calcium could be detected in the control samples, while those that had been loaded with BMP-2 contained mineralized extracellular matrix (Figure 10B). The amount of calcium measured within the UC scaffolds was clearly higher compared to the MC scaffolds, although the differences were only found to be statistically significant when BMP-2 used together with VEGF due to the high degree of variability inherent to these types of experiments.

## 4. Discussion

Biomaterials with organized microstructures can influence the orientation and behavior of the cells that grow on or inside of them, affecting the regeneration of target tissues [15,30,31]. Controlling the microstructure of ceramic, bioglass or synthetic polymer-based scaffolds has become relatively simple in the last years thanks to the development of sophisticated 3D printing technologies [32,33,34,35]. However, it remains more challenging for protein-based scaffolds and less studies have been performed with these biomaterials [15,36]. Since collagen type I is one of the main biomaterials used in BTE approaches [37], in this work, collagen type I scaffolds with oriented unidirectional pores were obtained through a controlled freezing method and were compared with scaffolds with randomly organized multidirectional pores. Their ability to guide bone growth when loaded with BMP-2 or BMP-2 + VEGF was evaluated, as well as the impact that implanted UC and MC scaffolds have on the amount of bone matrix formed in an ectopic rat model.

Porosity is well known to be a key parameter in BTE products, as it will determine the spatial distribution of cells, nutrients, oxygen and growth factors within the scaffold, directly affecting the viability of the bone that is being formed [35]. In both scaffolds, the pores were interconnected and the porosity was above 90%, allowing cells to adhere, grow and differentiate and for nutrients and waste products to diffuse properly [38].

The pore size of a scaffold must be determined according to the type of cells with which it will interact and the availability of ligands for efficient binding of cells to the biomaterial [20]. Scaffolds used for BTE usually have pore sizes in the range between 100 and 300 µm. Pores smaller than 100 µm might prevent the formation and growth of capillaries, leading to hypoxic conditions within the implants that will ultimately only allow the infiltration of fibrous tissue or, at best, a partial growth of unmineralized osteoid. Contrarily, larger pore sizes will permit blood vessels to grow and support bone formation [28,30]. The pore size of the two types of scaffolds evaluated in this work was very similar (with an average diameter of 260 to 268 µm) and allowed cell migration, adhesion and proliferation. In a previously published work, significant differences between the pore size of unidirectional and multidirectional pores of collagen type I scaffolds were found [20]. In this current work, however, the manufacturing procedure of the unidirectional scaffolds was modified by introducing a device that enclosed and cooled the environment in which the molds containing the collagen dispersion were frozen, by using a temperature gradient created with an aluminum foil immersed in liquid nitrogen. By enclosing the environment, the interaction between the surface of the collagen poured into the molds and the surrounding temperature was better controlled, leading to the similarity between the pore size of the unidirectional and multidirectional scaffolds found here.

Porosity is a parameter closely related and inversely proportional to the mechanical strength of a scaffold. Finding the best combination of porosity and mechanical properties in scaffolds for BTE remains a challenge [28]. In the present work, although the uni- and multidirectional scaffolds had the same porosity, the first had a significantly higher tensile strength than the multidirectional ones. A possible explanation for this is by the fact that, in the UC scaffolds, the load transmission falls mainly on the unidirectional pores, formed by the collagen fibers in the axial direction, counteracting the tensile forces. In contrast, the pores and fibers of MC scaffolds behave in a unitary way and must resist stress individually as they are not organized in a single direction. Other authors have obtained similar results when comparing the mechanical behavior of uni- and multidirectional porous hydroxyapatite scaffolds, showing that the axial direction of the pores contributes to the mechanical behavior [39]. Additionally, it has been shown that UC scaffolds have a higher modulus of elasticity than MC scaffolds, probably because the collagen fibers of these scaffolds act as cables that contribute to the elastic recovery of the material before reaching a plastic phase [38].

Both scaffolds had a similar degradation rate when incubated with collagenase, not exceeding a 15% degradation after 96 h. Since osteogenesis is a relatively slow process, the degradation rate of the scaffolds used for BTE must be in accordance with it [28]. The chemical crosslinking of the collagen fibers increased the resistance to enzymatic hydrolysis, making this biomaterial more suitable for BTE [13,24]. The final pore directionality within the sponges seemed to have no influence on this parameter.

Liquid uptake is another important parameter to consider when designing a scaffold for BTE, as water uptake and swelling will in part determine the absorption of body fluids and the transfer of cells, nutrients and metabolites within the scaffold. In addition, swelling of the microstructure increases the effective pore size and maximizes the internal surface area, hence influencing cell migration and adhesion. Under physiological conditions, however, swelling must be controlled, as an excess can cause weakening of the structure and its quick degradation [40]. In this work, the equilibrium state where the scaffolds cannot incorporate more liquid was reached in the first 10 min, highlighting the ability of these structures to act as sponges. The liquid sorption of the UC scaffolds was double that of the MC scaffolds because the parallel pores of UC act as channels that keep the liquid within the microstructure. The swelling dynamics of these scaffolds are comparable to those already reported [41].

In vitro tests using BM-MSCs clearly showed that both the UC and MC scaffolds are bioactive and able to support cell adhesion and growth, although the cells seem to have some more difficulties to adhere and start a proliferative phase in the early stages of the culture on the UC samples. Nevertheless, similar cell densities were obtained after 10 days with both types of scaffolds. Since the cell seeding was not forced by placing them directly on top of the scaffolds, but they were kept in suspension allowing them attach or not to the biomaterial, the lower seeding efficiency seen with the UC scaffold might simply be reflecting an initially lower accessible surface. As the cells grow on the scaffolds and remodel the superficial collagen layers, new pores might become accessible, allowing the cells to reach the same density as on the MC scaffolds after 10 days in culture. In any case, the behavior of the cells on the sponges, compared to the control scaffold, evidenced that these were biocompatible and retained no residual cytotoxicity from any of the manufacturing steps.

The ability of the cells to differentiate was assessed by measuring the ALP activity in the cultures in response to stimulation with BMP-2, VEGF or both. Surprisingly, the control cultures, without added growth factors, revealed a certain effect of the microstructure on the natural tendency towards differentiation of the cells when cultured on type I collagen scaffolds, as the ALP activity was significantly higher when cells grew on MC than on UC. This effect was maintained when BMP-2 alone was added to the cultures. As expected, this growth factor increased the ALP activity of the cells, but the final value was again significantly higher in the MC than in the UC scaffolds. Rather than a direct effect of the microstructure on cell differentiation, this might, again, just be reflecting differences in the available surface during the first days in culture. Although the same number of cells was seeded on both types of scaffolds, a more limited access to the interconnected network of pores in the UC sponges during the first days might be causing a lower seeding efficiency and, thus, a lower ALP activity after ten days in culture. Nevertheless, more in-depth cell physiology studies are needed to investigate to what extent the cells are functionally affected by the microstructure of these scaffolds.

VEGF alone did not induce osteogenic differentiation of the MSCs, but rather inhibited the ALP activity induced by BMP-2, as both growth factors act as signals that, oppositely, direct the cells towards the endothelial and osteogenic differentiation, respectively [16,17,18].

It is well known that osteogenesis is extremely difficult to mimic in vitro because it is a very complex multifactorial process. Therefore, the ability of a BTE construct to induce bone formation needs to be tested in vivo, as the relatively simple conditions of the in vitro assay are very different from those of the complex physical, chemical and cellular environment found in muscle pouches. We chose an ectopic model in rats as it allows for a fast and easy screening of the osteogenic properties of the scaffolds without interference of autogenous bone-related factors. We already knew from previous works that a dose of 500 ng of rhBMP-2 is slightly above the minimum dose required to trigger osteogenesis in ectopically implanted hemostatic collagen scaffolds [26]. With such dose, most of the implants should develop some degree of osteogenesis after 21 days. The scaffolds loaded with BMP-2 +VEGF were tested because osteogenesis is a process closely linked to angiogenesis and it has been extensively studied that the combination of BMPs with VEGF enhances bone formation [16,17,18]. Since a low dose of BMP-2 was used, a possible synergistical effect of another growth factor can be more easily detected.

The histological analysis of the implanted collagen scaffolds revealed, as expected, that the control and VEGF-loaded samples did not contain bone tissue after 21 days. However, the BMP-2 loaded implants contained variable amounts of bone tissue characterized by the presence of mature bone trabeculae with embedded osteocytes and cartilage remnants suggesting the endochondral origin of the newly formed bone, as frequently found in ectopic implants with BMPs [26,42]. Microscopic visualization of the implants suggested that more bone had formed within the UC scaffolds than in the MC scaffolds. Furthermore, the spatial orientation of the newly formed trabeculae seemed to be, at least in part, influenced by that of the collagen fibers in the microstructure. It was recently shown that the unidirectional orientation of collagen scaffold pores influences the formation of the bone matrix, directing it towards the endochondral pathway rather than the intramembranous one. This occurs because a unidirectional microarchitecture limits the formation of blood vessels and, consequently, the supply of oxygen favoring chondrogenesis [15]. The analysis of the calcium content within the implants further corroborated that the unidirectional orientation of the collagen fibers might have some positive effect on bone matrix formation, as significantly more mineral was found in the UC sponges loaded with BMP-2 and VEGF than on the MC implants. The fact that this happened in an ectopic osteogenesis model, where new bone is created without the influence of pre-existing osseous tissue, gives more insight on the importance of the microarchitecture of collagen scaffolds on tissue guidance.

The presence of rSp7 mRNA was greater in the implants loaded with BMP-2 and VEGF but did not differ between the MC and UC implants. Osterix is known to be one of the key transcription factors that regulate osteoblast differentiation and maturation [43]. Hence, its greater presence in the implants loaded with BMP-2 and VEGF indicates that there is still an active process of osteoblastic differentiation happening within these ectopic ossicles.

Although recently it has become clear that scaffold features such as pore geometry [30] and pore orientation [15] strongly affect cell behavior and bone formation, the factors that are ultimately responsible for the greater mineralization of the UC scaffolds remain unknown. In addition to the microstructure of the scaffolds other related features, such as the swelling capacity or the biomechanical properties, should be considered as these differ between the UC and MC. Future, in-depth studies on cell-collagen interactions or fluid and molecule dynamics would be needed to separately analyze each of these factors. In addition, although it was already demonstrated in previous works that VEGF can positively influence BMP-induced osteogenesis through different pathways [44], the exact crosstalk that occurs between these two growth factors during bone formation in vivo remains unclear and require more studies for being unraveled.

## 5. Conclusions

We found that the directionality of the pores within collagen type I scaffolds influenced their mechanical properties, as the UC sponges had a higher tensile strength and modulus of elasticity. Furthermore, the unidirectional disposition of the collagen fibers seemed to also affect in vivo ectopic osteogenesis, since a tendency of the bone trabeculae to follow the orientation of the pores was observed. Finally, a higher matrix mineralization was found in the UC scaffolds loaded with BMP-2 + VEGF. These findings on ectopic osteogenesis support previous works that studied the effect of pore directionality in other biomaterials and/or models. However, since most of these studies have been performed on stiffer, non-compressible materials, there is still a lack of information on how the directionality of pores within collagen scaffolds influence osteogenesis *in vivo*.

Although further in-depth studies are required to unravel how the microarchitecture of scaffolds affects cell behavior and can guide tissue growth, works like this one might be useful for the future design of more complex bone substitutes combining scaffolds with different features to better mimic the complexity of natural bone.

## Data Availability

The data presented in this study are available on request from the corresponding author.
